# Supplementary material for: A surface-confined spiral state with the double period in the cubic chiral helimagnet Cu2OSeO3
Source: NPJ Quantum Mater. 2026 May 21;11(1):86. doi: 10.1038/s41535-026-00892-6 (PMC13645675; doi:10.1038/s41535-026-00892-6)
Supplement: Supplementary file 1 [file 41535_2026_892_MOESM1_ESM.pdf]

Supplementary Materials

for

"A Surface-confined Spiral State With The Double Period In The Cubic Chiral Helimagnet Cu_2OSeO_3"

Priya R. Baral,[1,2,3,*] Oleg I. Utesov,[4] Samuel H. Moody,[2] Matthew T. Littlehales,[5,6] Pierluigi Gargiani,[7] Manuel Valvidares,[7] Robert Cubitt,[8] Nina-Juliane Steinke,[8] Chen Luo,[9] Florin Radu,[9] Arnaud Magrez,[3] Jonathan S. White,[2] and Victor Ukleev[9,†]

[1]Department of Applied Physics and Quantum-Phase Electronics Center (QPEC), The University of Tokyo, Bunkyo-ku, Tokyo 113-8656, Japan

[2]Laboratory for Neutron Scattering and Imaging (LNS), PSI Center for Neutron and Muon Sciences, Paul Scherrer Institute (PSI), CH-5232 Villigen, Switzerland

[3]Institute of Physics, École Polytechnique Fédérale de Lausanne (EPFL), CH-1015 Lausanne, Switzerland

[4]Center for Theoretical Physics of Complex Systems, Institute for Basic Science, Daejeon 34126, Republic of Korea

[5]Department of Physics, Durham University, Durham DH1 3LE, United Kingdom

[6]ISIS Neutron and Muon Source, Rutherford Appleton Laboratory, Didcot, OX11 0QX, United Kingdom

[7]ALBA Synchrotron Light Source, E-08290 Cerdanyola del Vallés, Barcelona Spain

[8]Institut Laue–Langevin, 71 avenue des Martyrs, CS 20156, Grenoble, 38042 Cedex 9, France

[9]Helmholtz-Zentrum Berlin für Materialien und Energie, D-12489 Berlin, Germany

[*]priya.baral@epfl.ch

[†]victor.ukleev@helmholtz-berlin.de

This supplementary material file contains supporting information for resonant elastic X-ray scattering (REXS) experiments, small-angle neutron scattering (SANS) experiments, and our analytical approach based on perturbation theory.

REXS EXPERIMENT

Experimental conditions and analysis of 1D data: Due to the limited angular size of the magnet window (90°), the measurement was performed in the imperfect Bragg condition. The ideal setting $2\theta = 96°$, $\theta = 48°$ was only used for the zero-field measurements with the magnet moved away from the sample. For the field scans we used the condition $2\theta = 88°$, $\theta = 45°$ in order to observe the REXS patterns on the tail of the Bragg peak. Such a relaxed setting is only allowed at the L-edge thanks to the limited penetration depth in the material, with the Bragg peak practically replaced with the crystal truncation rod (CTR).

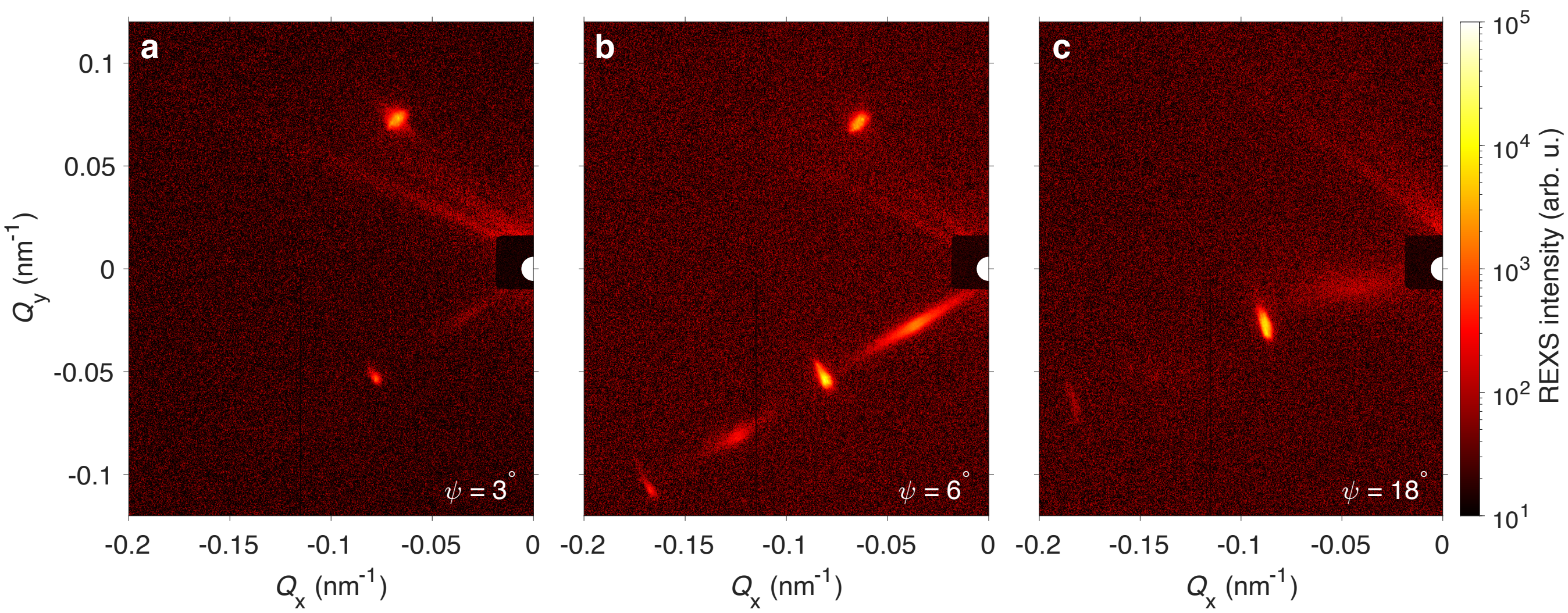

FIG. S1. **REXS at multiple azimuthal angles.** Panels-**(a)**-**(c)** show REXS patterns acquired as the Cu_2OSeO_3 disc-shaped crystal was rotated by $3°$, $6°$, and $18°$, away from [110], respectively. The magnetic field was maintained along the horizontal direction throughout the measurements. A broad, diffuse streak is observed towards high angle limit. All data shown here are collected at ~25 mT.

Two single crystals from two growth batches with distinct morphologies and surface treatments were employed in this study. The disc-shaped crystal served as the primary sample for obtaining the majority of key results, including both REXS and SANS measurements. The reproducibility of the SSS was independently verified on the rectangular-shaped crystal. For the REXS measurements on both crystals, low-angle Bragg peak tail was analysed, as described above. The disc-shaped Cu_2OSeO_3 single crystal was first aligned along [110]

horizontal axis ($\psi = 0°$), producing the four-spot characteristic REXS pattern from the in-plane ⟨100⟩ domains (Fig. 1f). We then performed a comprehensive azimuthal scan by rotating the sample in $3°$ steps across a range of $-51°$ to $+60°$, collecting REXS patterns at 5 mT intervals during field sweeps from 100 mT to 0 mT at each of the 38 angular positions. This sequence enabled us to track the evolution of field-polarised, conical, CSL and helical phases as the magnetic field was varied with respect to the crystallographic axes.

After first detecting the SSS at $\psi = 6°$, we reduced the angular resolution to $\Delta\psi = 1°$ and determined that the SSS stability window extends from $\psi = 3°$ to $\psi = 18°$ ($15°$ wide), with maximum intensity at $\psi = 9°$. Figure S1 shows REXS detector maps at two extreme azimuthal angle limits of the SSS stability ($\psi = 3°$ and $18°$) together with that obtained at $\psi = 6°$. To test reproducibility, we repeated the measurement protocol on the rectangular-shaped Cu_2OSeO_3 crystal and again observed the SSS with maximum intensity at $\psi = 9°$, demonstrating that the SSS is intrinsic and robust against variations in crystal morphologies, growth conditions, and surface preparations. Note that we observe weaker diffuse scattering at some azimuthal angles, which do not peak at $\mathbf{Q}_{\text{Hel}}/2$.

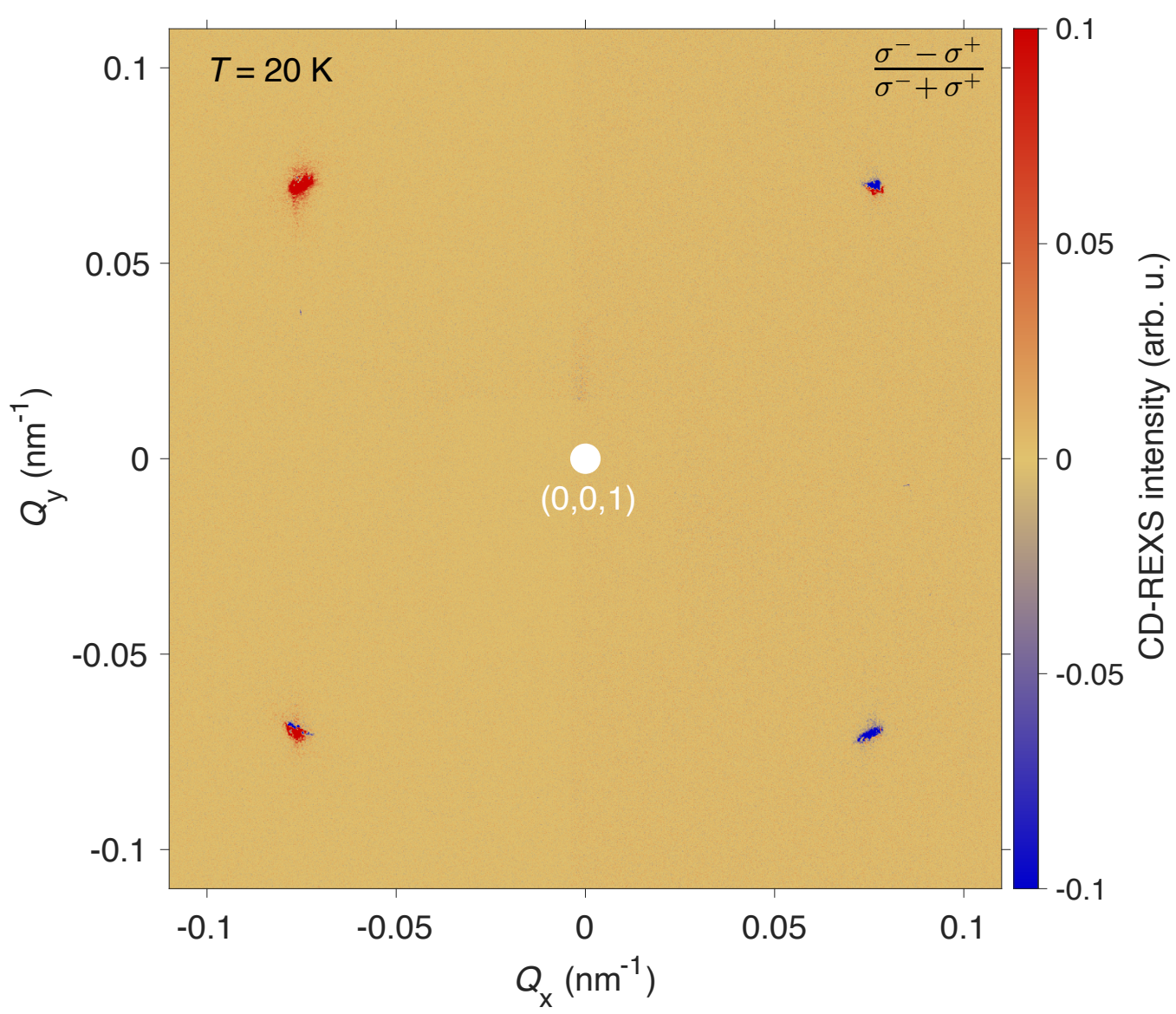

FIG. S2. **CD-REXS in helical state.** Normalised circular dichroism-REXS signal obtained from helical state at T = 20 K in the Cu_2OSeO_3 disc sample.

One-dimensional intensity profiles as a function of momentum transfer $\mathbf{Q}$ were extracted from the two-dimensional detector maps and subsequently fitted to the following functional form:

$$y = y_0 + \frac{A}{w\sqrt{(\pi/(4\ln 2))}} e^{\left(\frac{-4\ln 2(x-x_c)^2}{w^2}\right)} + A_1 e^{-x/t_1}, \tag{S1}$$

where, y_0, A_1, t_1 are constants, A is the area under the fitted curve, w is the width of the fit and x_c is the center of the peak. Results of these fits are shown in Fig. 3.

CD-REXS: Circular dichroism in resonant elastic X-ray scattering (CD-REXS) determines both the chirality of the modulated magnetic textures, and their Bloch-type or Néel-type character. Prior to determining the chirality of SSS, we first performed CD-REXS measurements on the zero-field helical state at 20 K. These results are presented in Fig. S2. Reflections within individual Friedel pairs exhibit opposite dichroic signals, a direct consequence of the underlying chirality of the magnetic structure. Furthermore, we identify the near-vertical direction in the detector plane as the *extinction direction*, where dichroic signal vanishes.

SCANNING-REXS EXPERIMENT

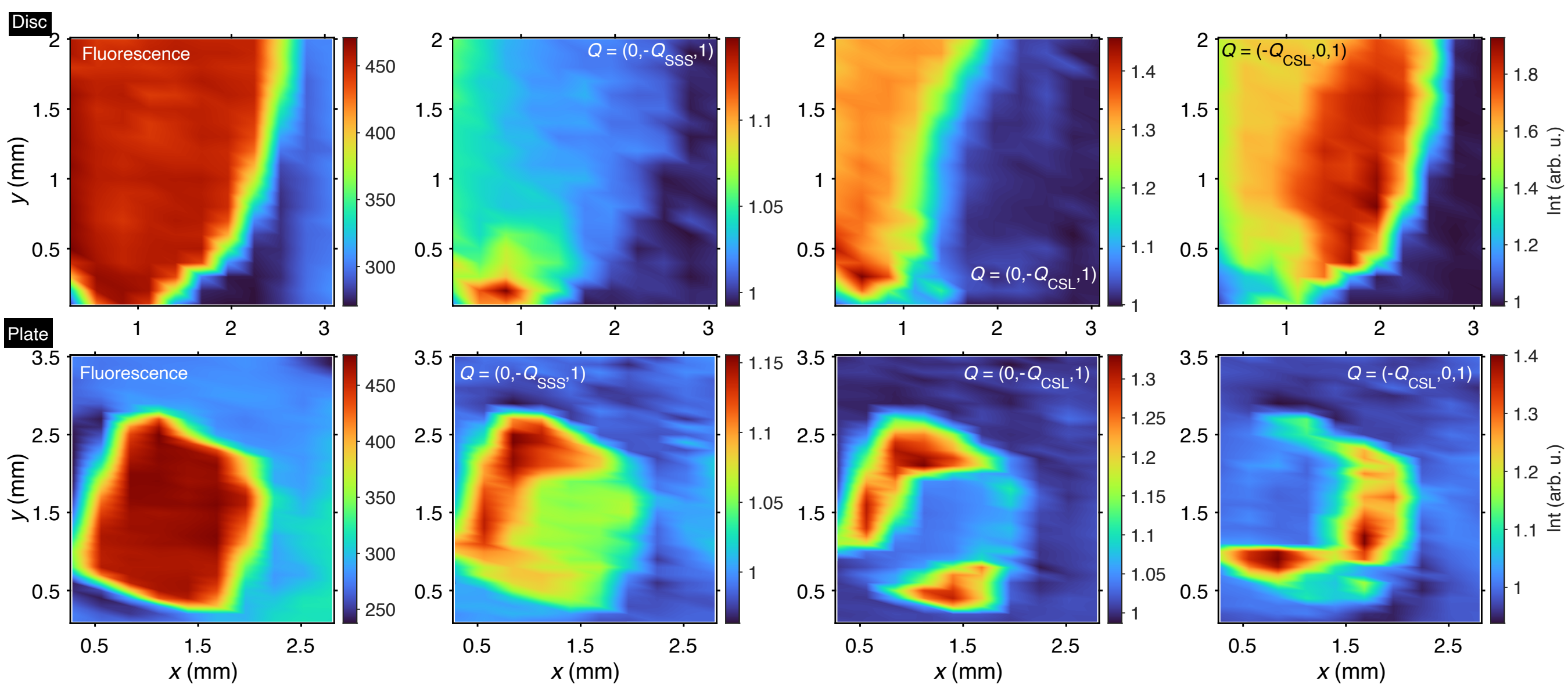

FIG. S3. **REXS probing larger portion of the surface.** Scanning-REXS maps of the circular disk and the rectangular plate of Cu_2OSeO_3 single crystal sample used in this study. Note that the spatial distribution of the SSS correlates systematically with the CSL domain closer to external magnetic field, that is $\mathbf{Q} = (0, \pm\mathbf{Q}_{\mathrm{CSL}}, 1)$. Full surface of the disc-shaped crystal is not mapped. We observe a more prominent presence of the SSS in the rectangular crystal.

Scanning-REXS experiment was performed to map the SSS on two samples: a Cu_2OSeO_3 disc with diameter of 5 mm and a plate ca. 2×2 mm (see Supplementary Fig. S3). The intensity of two CSL peaks, corresponding to modulations aligned with (100) and (010), and SSS peak ($\mathbf{Q}_{\mathrm{SSS}}$) were traced as a function of the sample position in the (x, y) plane. Total fluorescence was measured as a background signal. The x-ray spot size on the sample surface at $\theta = 45°$ was 100 μm in vertical and 280 μm in horizontal directions, respectively. Note, that in case of the disc the scan did not cover the whole surface.

Scanning-REXS maps of a Cu_2OSeO_3 disc are shown in Fig. S3. Evidently, the sample shows distinct regions of single-domain CSL textures aligned in orthogonal cubic axes, and also regions with co-existing CSL

domains. Notably, the SSS state occupies the largest fraction in a narrow region close to the bottom edge of the disc. The separation into CSL domains is more prominent in case of the plate-shaped sample, where also the SSS occupies larger fraction of the surface area Fig. S3.

SMALL-ANGLE X-RAY SCATTERING

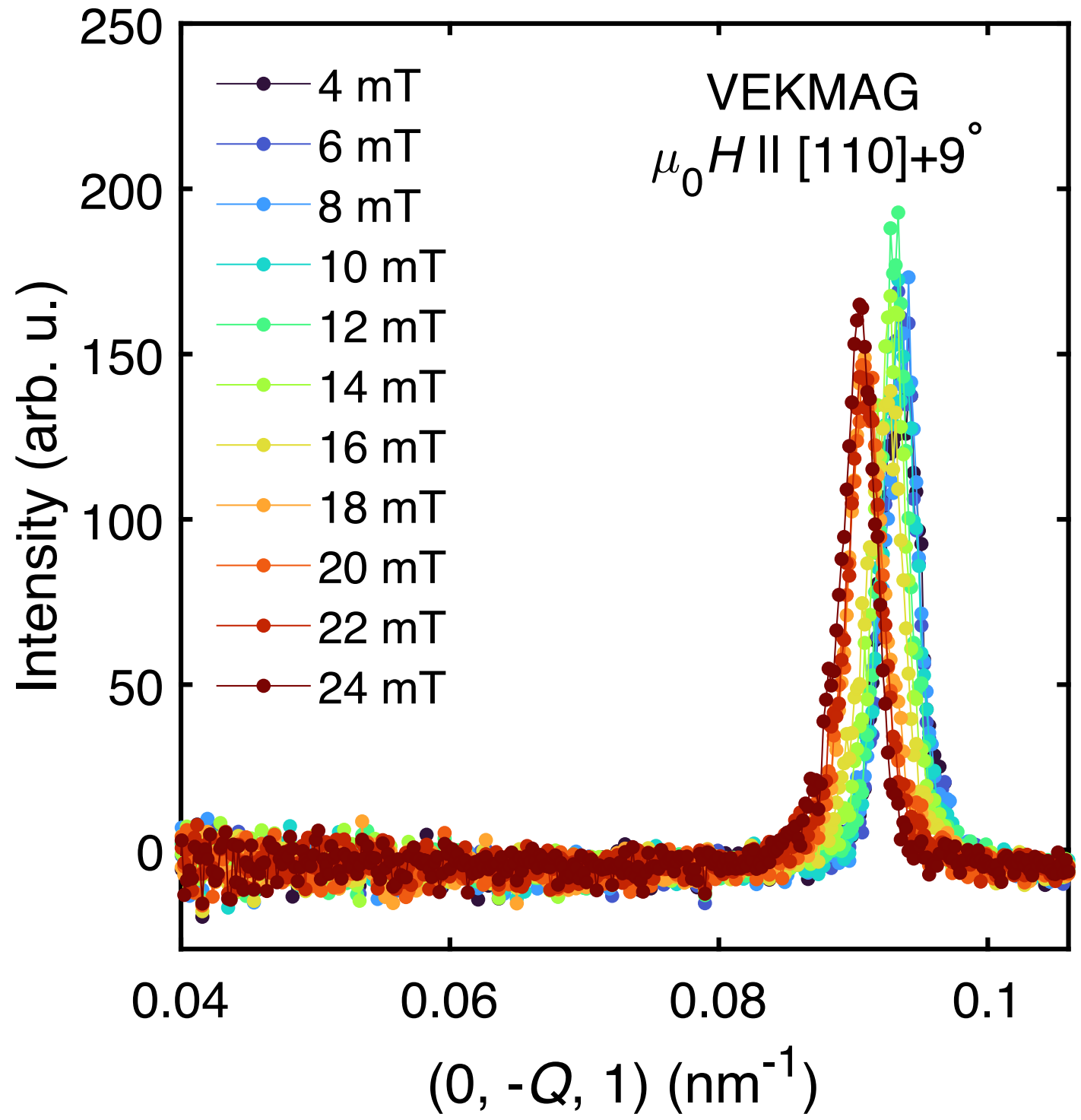

FIG. S4. **Comparing REXS with SAXS.** Integrated intensity of SAXS signals measured on a (001) Cu_2OSeO_3 lamella at VEKMAG. The measurements are taken at $T = 14$ K and with the field applied along $\langle 110 \rangle$ by 9° in the sample plane. The Bragg peak from conical and CSL states is observed at $\mathbf{Q} \approx 0.09$ nm^{-1}. No signature of the $\mathbf{Q}_{\mathrm{SSS}}$ at $\mathbf{Q} \approx 0.045$ nm^{-1} is seen.

Small-angle resonant x-ray scattering (SAXS) measurements were performed on a thin Cu_2OSeO_3 lamella prepared by focused ion beam (FIB) from a bulk single crystal, with the lamella surface-normal oriented along the [001] crystallographic direction. The lamella was mechanically fixed to a gold-coated Si_3N_4 membrane (Silson Ltd, UK) using two Pt contacts deposited via FIB. Prior to mounting, a 5×5 μm^2 square aperture was milled through both the membrane and gold layer to enable X-ray transmission through the sample. Detailed fabrication procedures can be found in Supplementary Ref. [1]. The lamella was mounted on a Cu sample holder and inserted into a vector-field cryomagnet (VEKMAG) installed at the PM-2 beamline of BESSY II (Berlin, Germany). The circularly polarised soft X-ray beam energy was tuned to 929.5 eV (Cu L_3-edge) to

maximise the intensity of magnetic satellites. Following zero-field cooling to a base temperature of 14 K, SAXS measurements were performed using a vector-field geometry. To achieve faster frame rates without sacrificing angular resolution, binning by a factor of 2 was applied. Typical acquisition times for each small-angle diffraction pattern were 60 seconds.

At base temperature (14 K), we performed scans while ramping magnetic field up and down. Neither measurements reveal any sign of $\mathbf{Q}_{\mathrm{SSS}}$. Part of these scans are shown in the Fig. S4. We also checked for appearance of SSS up to T_{C}. Further information can be found in Supplementary Ref. 1.

SMALL-ANGLE NEUTRON SCATTERING

Multiple magnetic Bragg peaks are observed in SANS patterns at the helical-to-conical transition corresponding to helicoidal [$(\pm\mathbf{Q}_{\mathrm{CSL}}, 0, 0)$ and $(0, \pm\mathbf{Q}_{\mathrm{CSL}}, 0)$] and conical ($\pm\mathbf{Q}_{\mathrm{con}}$) textures, and their higher harmonics. Importantly, lower-Q peaks that emerge in both SANS configurations (Fig. S5**a**,**c**) correspond to multiple scattering processes [$(\pm\mathbf{Q}_{\mathrm{CSL}}, \mp\mathbf{Q}_{\mathrm{con}}, 0)$], rather than SANS from a distinct phase. Detailed field-dependent radial profiles of SANS patterns are shown in Figs. S5**b**,**d**. They show no indication of the $\mathbf{Q}_{\mathrm{SSS}}$ peak; therefore, consistent with the surface nature of the new state.

THEORETICAL ESTIMATIONS

One of the experimental hints allowing us to speculate about the nature of the SSS spin structure is the absence of the corresponding second harmonic peak and the significant intensity of the third-order one on the REXS maps. The estimations below are based on the well-studied sine-Gordon equation model for helical structures subject to low-symmetry perturbations.

In the case of magnetic field transverse to the modulation vector, the helicoid phase is described by the following functional:

$$F[\varphi] = \frac{1}{L}\int dz \left[\frac{1}{2}\left(\frac{d\varphi}{dz}\right)^2 - q\frac{d\varphi}{dz} - \alpha\cos\varphi\right], \tag{S2}$$

where the first term corresponds to exchange coupling, the second one to DMI, and the third to Zeeman energy. Whereas, $q = D/J$ is the spiral wave number at zero field, $\alpha = h/SJ$ is the dimensionless magnetic field, and L is the structure period. This functional should be minimised with respect to $\varphi(z)$. The result is well-known (see, e.g., Supplementary Refs. 2,3,4): crucial parameter m satisfies the equation

$$\frac{4E(m)}{\pi q} = \sqrt{\frac{m}{\alpha}}, \tag{S3}$$

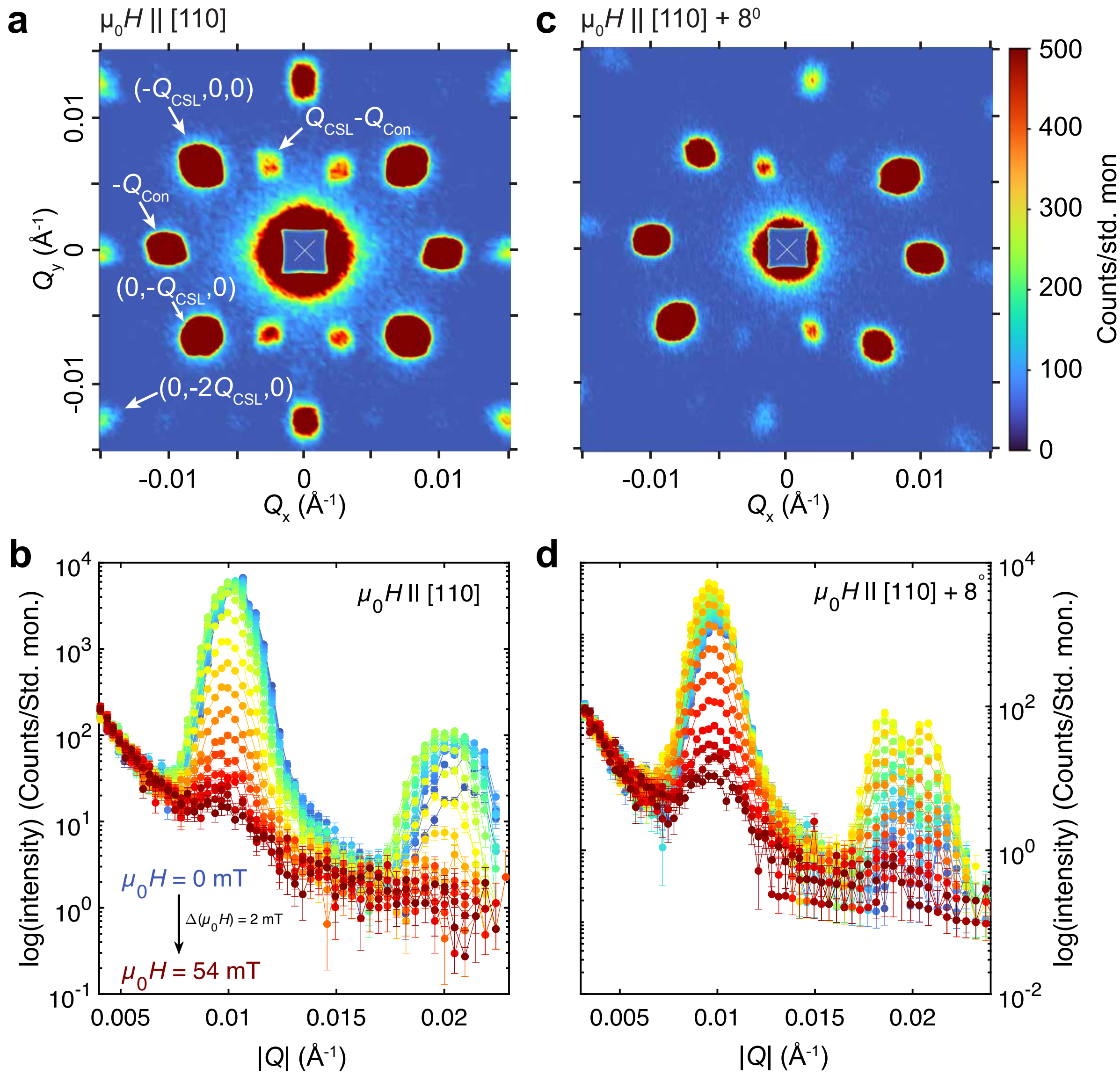

FIG. S5. **Small angle neutron scattering results on Cu_2OSeO_3 single crystal.** **(a)** shows the resultant detector image at a finite field ($\mu_0 H$ = 36 mT ‖ [110]) with coexistence of CSL phase with conical phase and their respective higher harmonic reflections. Reflections arising due to multiple scattering are also labelled. **(b)** shows the 1D line scans through CSL (and its higher harmonic) reflection as a function of magnetic field. **(c)** shows the detector map for $\mu_0 H$ ‖ [110] + 8° at 34 mT. Similar line scans presented in Panel-**(d)** rule out the presence of SSS in bulk Cu_2OSeO_3.

where $E(m)$ is the complete elliptic integral of the second kind, the phase is expressed via the Jacobi amplitude function

$$\varphi(z) = 2\,\mathrm{am}\left(\frac{\pi q z}{4E(m)}, m\right) + \pi, \tag{S4}$$

and the helicoid period reads

$$L_{kink} = \frac{8K(m)E(m)}{\pi q}. \tag{S5}$$

Here, $K(m)$ is the complete elliptic integral of the first kind. Importantly, at $\alpha \to \alpha_c \equiv (\pi q/4)^2$ the structure period diverges, manifesting a transition to the field-polarised phase (Supplementary Ref. 5).

For illustration purposes, we consider the magnetic field corresponding to the appearance of the SSS structure, i.e., $h \approx 0.25 h_{C2}$, and the CSL modulation vector at an angle $\approx 45^\circ$ with respect to the field direction. Then, $\alpha/\alpha_c = 0.3$ and the in-plane spin component $\approx 0.97S$. The latter allows us to use the model of the co-planar CSL presented above for estimations. Eq. (S3) yields $m \approx 0.53$, so the period of the structure is only slightly larger than the one at $h = 0$ $[L(h = 0) = 2\pi/q]$ being $L \approx 1.02L(h = 0)$. Moreover, the higher harmonics are also small. For example, the second-order ones have the relative amplitude

$$\frac{S_2^{x,y}}{S_1^{x,y}} \approx \frac{0.047S}{0.497S} = 0.09. \tag{S6}$$

The amplitudes of third-order harmonics are about ten times weaker, etc.

For the SSS structure, we have not observed any traces of the second harmonic. Thus, we believe that it can emerge due to effective axial anisotropy at the surface. To treat this case, one should modify the equations above accordingly. The functional subject to minimisation now reads

$$F[\phi] = \frac{1}{4L} \int dz \left[\frac{1}{2} \left(\frac{d\phi}{dz} \right)^2 - 2q \frac{d\phi}{dz} - \frac{2B}{J} \cos\phi \right], \tag{S7}$$

where B measures easy-axis anisotropy and the angle of the spin in real space φ is related to variable ϕ as $2\varphi = \phi$. So, the problem is equivalent to the previous one, but with alterations $\tilde{q} = 2q$ and $\tilde{\alpha} = 2B/J$. Thus we have

$$\varphi(z) = \text{am}\left(\frac{\pi q z}{2E(m)}, m \right) + \frac{\pi}{2}, \tag{S8}$$

The double period of the structure in this case corresponds to a strongly modulated order (see Fig. 3) with $m \approx 0.999$. The corresponding harmonic amplitudes are

$$S_1^x \approx 0.61S,\ S_1^y \approx 0.28S,\ S_3^x \approx 0.15S,\ S_3^y \approx 0.14S, \tag{S9}$$

whereas the second-order harmonics are negligible. We conclude that the relative intensity of the third-order peak in this scenario is significant, which supports the idea that SSS is a strongly-modulated stripe structure.

We note that the value of m used here seems to be fine-tuned. In fact, to prove the proposed scenario of SSS appearance, a detailed microscopic theory is required. However, we would like to point out that the nearly critical anisotropy can be relevant to the fact that in the case of the thin lamella, SSS was not observed. Evidently, small alterations of the anisotropy (e.g., by the tension of the lamella or due to the dipolar field in the quasi-2D case) can make it stronger, favoring collinear structures instead of modulated ones.

SUPPLEMENTARY REFERENCES

S[1] P. R. Baral, O. I. Utesov, C. Luo, F. Radu, A. Magrez, J. S. White, and V. Ukleev, Direct observation of exchange anisotropy in the helimagnetic insulator Cu_2OSeO_3, Physical Review Research **5**, L032019 (2023).

S[2] I. E. Dzyaloshinskii, Theory of helicoidal structures in antiferromagnets. III, Sov. Phys. JETP **20**, 665 (1965).

S[3] J-I. Kishine, and A.S. Ovchinnikov, Theory of Monoaxial Chiral Helimagnet, Solid State Physics **66**, 1 (2015).

S[4] Y. A. Izyumov, and V. M. Laptev, Neutron diffraction by incommensurate magnetic structures, Soviet Journal of Experimental and Theoretical Physics **58**, 1267 (1983).

S[5] V. Laliena, J. Campo, and Y. Kousaka, Nucleation, instability, and discontinuous phase transitions in monoaxial helimagnets with oblique fields, Phys. Rev. B **95**, 224410 (2017).
